# Supplementary material for: ß-adrenergic-like signalling engages CrebB in Drosophila gut to promote female longevity
Source: Nat Commun. 2026 Apr 2;17:4844. doi: 10.1038/s41467-026-71341-y (PMC13222880; doi:10.1038/s41467-026-71341-y)
Supplement: Supplementary file 1 — Supplementary Information [file 41467_2026_71341_MOESM1_ESM.pdf]

a *LM* for TA level in *Tdc2>Tdc2* flies
Call: lm(formula = Conc. ~ Genotype + Sex + Genotype:Sex)

Coefficients	Estimate	Std.Error	T value	Pr(>|t|)
Intercept	27.116	3.542	7.655	3.21e−07
Genotype (Driven vs controls)	10.239	2.505	4.088	***0.000627
Genotype (*GAL4* vs *UAS)*	−6.745	4.339	−1.555	0.136537
Sex (Male)	−20.882	4.926	−4.239	0.000444
Genotype (Driven vs controls): Sex (Male)	−9.109	3.422	−2.662	0.015410
Genotype (*GAL4* vs *UAS)*: Sex (Male)	5.775	6.136	0.941	0.358397

b *LM* for OA level in *Tdc2>Tdc2* flies
Call: lm(formula = Conc. ~ Genotype + Sex + Genotype:Sex)

Coefficients	Estimate	Std.Error	T value	Pr(>|t|)
Intercept	44.494	8.302	5.359	***2.22e−05
Genotype (Driven vs controls)	8.151	5.870	1.388	0.179
Genotype (*GAL4* vs *UAS)*	−10.863	10.168	−1.068	0.297
Sex (Male)	−10.146	11.000	−0.922	0.366
Genotype (Driven vs controls): Sex (Male)	−8.394	7.827	−1.072	0.295
Genotype (*GAL4* vs *UAS)*: Sex (Male)	11.153	13.387	0.833	0.414

c *CPH* model for the lifespan of *Tdc2>Tdc2* flies
Call: coxph(formula = Surv(time = day, event = event) ~ Genotype + Sex + Genotype:Sex)

	coef	Exp.coef	Pr(>|z|)
Genotype (Driven vs controls)	−0.44416	0.64136	***< 2e−16
Genotype (*GAL4* vs *UAS)*	−0.32403	0.72323	***3.39e−08
Sex (Male)	1.19167	3.29258	***< 2e−16
Genotype (Driven vs controls): Sex (Male)	−0.03113	0.96935	0.55172
Genotype (*GAL4* vs *UAS)*: Sex (Male)	0.23724	1.26774	**0.00491

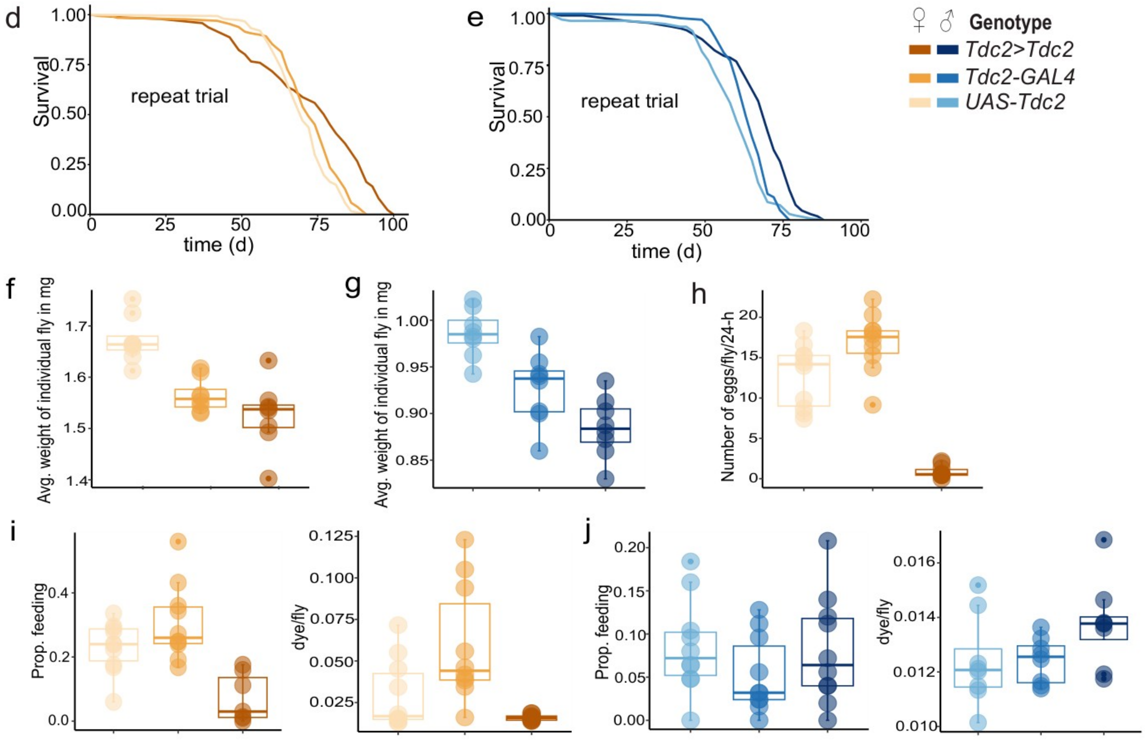

k *LM* for pAKT/AKT level in *Tdc2>Tdc2* flies
Call: lm(formula = Conc. ~ Genotype)

Coefficients	Estimate	Std.Error	T value	Pr(>|t|)
Intercept	1.00	0.07875	12.698	1.99e−09 ***
Genotype (Driven vs controls)	−0.06686	0.05569	−1.201	0.2485
Genotype (*GAL4* vs *UAS)*	−0.18960	0.09645	−1.966	0.0681

LM for pERK/ERK level in *Tdc2>Tdc2* flies
Call: lm(formula = Conc. ~ Genotype)

Coefficients	Estimate	Std.Error	T value	Pr(>|t|)
Intercept	1.00	0.04796	20.850	1.72e−12 ***
Genotype (Driven vs controls)	0.089498	0.033914	2.639	0.0186 *
Genotype (*GAL4* vs *UAS)*	0.009367	0.058740	0.159	0.8754

Supplementary Figure 1 *Tdc2* – lifespan and other phenotypes.
a-b, Summary of Linear Model (*LM*) analysis of **a,** TA and **b,** OA levels in *Tdc2>Tdc2* flies (data presented in **Figure 1b** and **c**). **c,** Cox Proportional Hazards (*CPH*) summary of *Tdc2>Tdc2* lifespan assays (survival presented in **Figure 1d**). **d-e,** Lifespan of *Tdc2>Tdc2* flies (repeat trial). *Tdc2>Tdc2*: n= 138 dead/4 censored females and n=110/4 males, *UAS-Tdc2*: n=144/5 females, n=130/9 males, *Tdc2-*

GAL4: n=105/26 females, n=140/3 males; significant effect of genotype (driven *versus* controls, $p=1.3x10^{-9}$) and sex ($p= 2x10^{-16}$), *CPH*. **f-g,** Weight measurements of *Tdc2>Tdc2* flies. n=8 biologically independent samples; significant effect of genotype (driven *versus* controls, p=$7.3x10^{-6}$) and sex (p=$2x10^{-16}$), *LM*. **h,** Fecundity levels of *Tdc2>Tdc2* flies. n=10 biologically independent samples; significant effect of genotype (driven *versus* controls, $p=5.1x10^{-12}$), *LM*. **i-j,** Proboscis extension (left) and food (dye) consumption (right) assays of **i,** female and **j,** male *Tdc2>Tdc2* flies. Biologically independent samples in proboscis extension assay: females, n = 7 (*Tdc2>Tdc2*), 8 (*Tdc2-GAL4*), 10 (*UAS-Tdc2*); males, n=10; food (dye) consumption assay: females, n = 10; males, n= 8. Probiscis extension assay: significant effect of genotype (driven *versus* controls, $p=5.9x10^{-5}$), sex ($p=2.7x10^{-6}$) and genotype-by-sex interaction (p=$4.4x10^{-4}$), *Generalized Linear Model* (*GLM*) with quasi-binomial distribution; food consumption assay: significant effect of genotype (driven *versus* controls, $p=1.36x10^{-4}$), sex ($p=3.83x10^{-5}$) and genotype-by-sex interaction (*p=0.02*), *LM*. **k,** Summary of *LM* analysis of pAKT/AKT and pERK/ERK levels in *Tdc2>Tdc2* flies (data presented in **Figure 1e** and **f**). **f**, **g**, **h, i,** and **j** boxplots show quantiles with individual data points overlayed. Where relevant, statistical tests were two-sided; no multiple testing correction was applied.

a *LM* for climbing data in *Tdc2>Tdc2* flies

Call:lm(formula = Height ~ Genotype + Time (d) + Sex + Genotype:Time + Time:Sex + Genotype:Sex:Time)

Coefficients	Estimate	Std.Error	T value	Pr(>|t|)
Intercept	15.100505	0.207120	72.907	***< 2e−16
Genotype (Driven vs controls)	−1.739039	0.111019	−15.664	***< 2e−16
Genotype (*GAL4* vs *UAS)*	−0.804830	0.172164	−4.675	***3.05e−06
Time (d)	−0.265738	0.006723	−39.524	***< 2e−16
Sex (Male)	1.311025	0.295099	4.443	***9.14e−06
Genotype (Driven vs controls): Time (d)	0.051564	0.003893	13.247	***< 2e−16
Genotype (*GAL4* vs *UAS*): Time (d)	0.017891	0.006203	2.884	**0.00394
Time (d): Sex (Male)	−0.031288	0.009815	−3.188	**0.00145
Genotype (Driven vs controls): Time (d): Sex (Male)	0.002055	0.003473	0.592	0.55399
Genotype (*GAL4* vs *UAS*): Time (d): Sex (Male)	−0.011490	0.005413	−2.123	*0.03386

b Ordinal regression model for Smurf assay in *Tdc2>Tdc2* flies

formula: Smurf ~ Genotype + Time + Sex + Genotype: Time + Genotype: Sex + Time: Sex

Coefficients	Estimate	Std.Error	T value	Pr(>|t|)
Genotype (Driven vs controls)	0.482527	0.331762	1.454	0.145825
Genotype (*GAL4* vs *UAS)*	−0.716688	0.427538	−1.676	0.093677
Time (d)	0.109221	0.009331	11.705	< 2e−16 ***
Sex (Male)	1.419947	0.671123	2.116	0.034364 *
Genotype (Driven vs controls): Time (d)	−0.016217	0.006115	−2.652	0.008001 **
Genotype (*GAL4* vs *UAS*): Time (d)	0.007162	0.007319	0.978	0.327836
Genotype (Driven vs controls): Sex (Male)	0.034767	0.178686	0.195	0.845728
Genotype (*GAL4* vs *UAS*): Sex (Male)	0.536375	0.162365	3.304	0.000955 ***
Time (d): Sex (Male)	−0.022426	0.012960	-1.730	0.083550

c

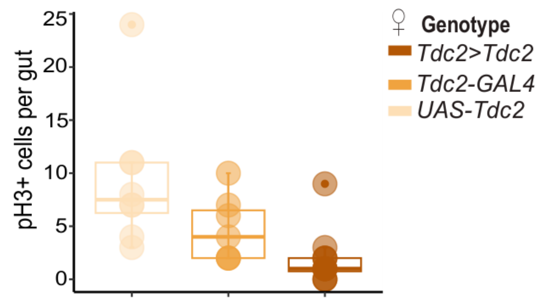

Supplementary Figure 2 *Tdc2* – health and other phenotypes.

a, *LM* summary - *Tdc2>Tdc2* climbing (data shown in **Figure 2a**). **b,** *Ordinal Logistic regression* summary - *Tdc2>Tdc2* gut barrier assay (Smurf assay; data shown in **Figure 2b**). **c,** Number of pH3+ cells per gut of *Tdc2>Tdc2* 63-day old female flies. Biologically independent samples: n = 12 (*Tdc2>Tdc2*), 7 (*Tdc2-GAL4*), 8 (*UAS-Tdc2*); significant effect of genotype (driven *versus* controls, p=$3.9x10^{-3}$), *LM*. Boxplot shows quantiles with individual data points overlayed. Where relevant, statistical tests were two-sided; no multiple testing correction was applied.

a *LM* for OA level in *Tbh>Tbh* flies

Call: lm(formula = Conc. ~ Genotype + Sex + Genotype:Sex)

Coefficients	Estimate	Std.Error	T value	Pr(>|t|)
Intercept	25.113	2.312	10.860	***9.59e−11
Genotype (Driven vs controls)	1.122	1.635	0.686	0.499
Genotype (*GAL4* vs *UAS)*	−1.317	2.832	−0.465	0.646
Sex (Male)	29.739	3.270	9.094	***3.04e−09
Genotype (Driven vs controls): Sex (Male)	−2.968	2.312	−1.283	0.212
Genotype (*GAL4* vs *UAS)*: Sex (Male)	−2.665	4.005	−0.665	0.512

b

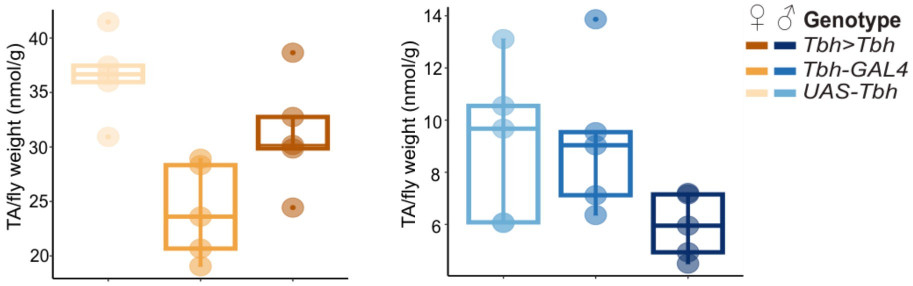

c *LM* for TA level in *Tbh>Tbh* flies

Call: lm(formula = Conc ~ Genotype + Sex + Genotype:Sex)

Coefficients	Estimate	Std.Error	T value	Pr(>|t|)
Intercept	30.5989	0.9424	32.470	***< 2e−16
Genotype (Driven vs controls)	0.2901	0.6664	0.435	0.667176
Genotype (*GAL4* vs *UAS)*	−6.1921	1.1542	−5.365	***1.65e−05
Sex (Male)	−22.5239	1.3327	−16.901	7.87e−15
Genotype (Driven vs controls): Sex (Male)	−1.3534	0.9424	−1.436	0.163871
Genotype (*GAL4* vs *UAS)*: Sex (Male)	6.2387	1.6322	3.822	***0.000825

d *CPH* model for the lifespan of *Tbh>Tbh* flies

Call: coxph(formula = Surv(time = day, event = event) ~ Genotype + Sex + Genotype:Sex)

	coef	Exp.coef	Pr(>|z|)
Genotype (Driven vs controls)	0.05879	1.06055	0.1017
Genotype (*GAL4* vs *UAS)*	−0.24997	0.77882	***5.09e−05
Sex (Male)	2.01511	7.50159	***< 2e−16
Genotype (Driven vs controls): Sex (Male)	−0.28334	0.75326	***3.53e−08
Genotype (*GAL4* vs *UAS)*: Sex (Male)	0.19462	1.21485	*0.0232

Supplementary Figure 3 *Tbh* – lifespan and other phenotypes.

a, *LM* summary - OA levels in *Tbh>Tbh* flies (data shown in **Figure 3b**). **b,** TA levels in *Tbh>Tbh* flies. n=5 biologically independent samples **c,** *LM* summary - TA levels in *Tbh>Tbh* flies. **d,** *CPH* summary- *Tbh>Tbh* lifespan (survival presented in **Figure 3c**). b, boxplots show quantiles with individual data points overlayed. Where relevant, statistical tests were two-sided; no multiple testing correction was applied.

a *LM* for TA level in TA and OA-fed flies
Call: lm(formula = Conc. ~ Food+ Sex + Condition:Sex)

Coefficients	Estimate	Std.Error	T value	Pr(>|t|)
Intercept	12.85747	0.69151	18.593	***9.31e−16
Food (TA vs control)	3.58389	1.00334	3.572	**0.00154
Food (OA vs control)	−1.73302	0.92504	−1.873	0.07323
Sex (Male)	0.49070	1.04632	0.469	0.64331
Food (TA vs control): Sex (Male)	0.89132	1.51127	0.590	0.56085
Food (OA vs control): Sex (Male)	0.07552	1.41450	0.053	0.95786

b *LM* for OA level in TA and OA-fed flies
Call: lm(formula = Conc. ~ Food+ Sex + Food:Sex)

Coefficients	Estimate	Std.Error	T value	Pr(>|t|)
Intercept	25.256	1.043	24.223	***< 2e−16
Food (TA vs control)	−2.517	1.469	−1.713	0.097101
Food (OA vs control)	3.822	1.529	2.500	*0.018136
Sex (Male)	7.207	1.609	4.479	***0.000101
Food (TA vs control): Sex (Male)	4.693	2.272	2.066	*0.047597
Food (OA vs control): Sex (Male)	−3.702	2.311	−1.602	0.119627

c *CPH* model for the lifespan of TA-fed flies
Call: coxph(formula = Surv(time = day, event = event) ~ TA (μg/ml) * Sex)

	coef	Exp.coef	Pr(>|z|)
TA (μg/ml)	−0.009030	0.991010	***9.12e−16
Sex (Male)	0.318436	1.374976	***3.92e−05
TA (μg/ml): Sex (Male)	0.009610	1.009656	***3.10e−10

d

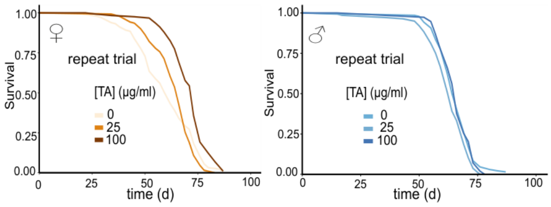

e

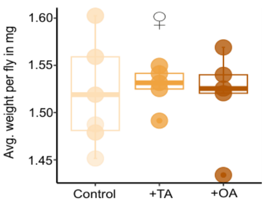

f

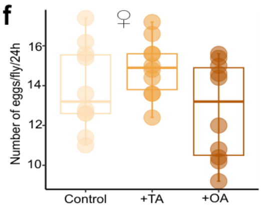

g

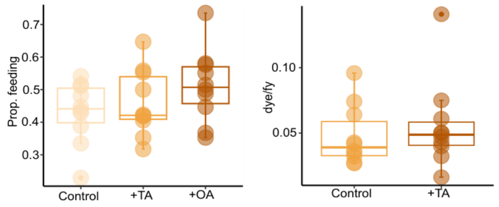

h *LM* for climbing data in TA-fed female flies
Call: lm(formula = Height ~ Food + time + Food: time)

Coefficients	Estimate	Std.Error	T value	Pr(>|t|)
Intercept	9.80591	0.38604	25.402	<2e−16 ***
Food (TA vs Control)	-0.93344	0.54548	-1.711	0.0874
Time (d)	-0.20276	0.01391	-14.573	<2e−16 ***
Food (TA vs Control): time (d)	0.04118	0.01965	2.095	0.0364*

i *Ordinal regression model* for Smurf assay in TA-fed female flies
formula = Smurf ~ Food + time

Coefficients	Estimate	Std.Error	z value	Pr(>|z|)
Food (TA vs Control)	−1.61521	0.33251	−4.858	1.19e−06 ***
Time (d)	0.20539	0.03162	6.496	8.22e−11 ***

j *CPH* model for the lifespan of OA-fed flies
Call: coxph(formula = Surv(time = day, event = event) ~ OA (μg/ml) * Sex)

	coef	Exp.coef	Pr(>|z|)
OA (μg/ml)	0.009587	1.009633	*0.039998
Sex (Male)	1.900158	6.686949	***< 2e−16
OA (μg/ml): Sex (Male)	−0.025820	0.974510	***0.000105

k

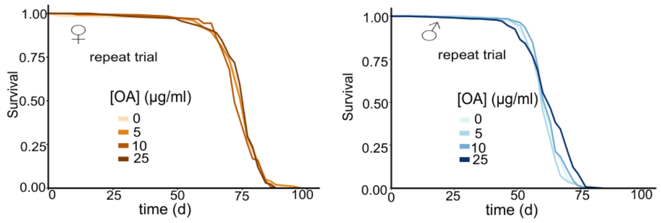

Supplementary Figure 4 TA and OA feeding.

a, *LM* summary - TA levels in TA and OA-fed flies (data shown in **Figure 4a**). **b,** *LM* summary - OA levels in TA and OA-fed flies (data shown in **Figure 4b**). **c,** *CPH* summary - lifespan of TA-fed flies (survival presented in **Figure 4c**). **d,** Lifespan of TA-fed flies (repeat trial). Control: n=142 dead/6 censored females and n=133/7 males; 25 μg/ml TA: n=138/2 females and n=130/8 males; 100 μg/ml TA: n=114/3 females and 106/12 males; significant effect of TA feeding (*p=1.74x10^{-9}*), TA feeding-by-sex interaction (*p=2.8x10^{-5}*), *CPH*. **e,** Weight measurements of TA and OA-fed female flies. n=10 biologically independent samples; effect of TA feeding (*p=0.86*), OA feeding (*p=0.59*), *LM*. **f,** Fecundity levels in TA and OA-fed female flies. n=10 biologically independent samples; effect of TA feeding (*p=0.28*), OA feeding (*p=0.24*), *LM*. **g,**

Proboscis extension (right) and food (dye) consumption (left) assay of TA and OA-fed female flies. n=10 biologically independent samples; proboscis extension assay: effect of TA feeding (*p=0.17*), OA feeding (*p=0.11*), *GLM* with quasi-binomial distribution; food (dye) consumption assay: effect of TA feeding (*p=0.54*), *t-test*. **h,** *LM* summary - TA-fed climbing (data shown in **Figure 4d**). **i,** *LM* summary - TA-fed gut barrier assay (Smurf assay; data shown in **Figure 4e**). **j,** *CPH* summary - OA-fed lifespan (survival presented in **Figure 4f**). **k,** Lifespan of OA-fed flies (repeat trial). Control: n= 133/5 females and n=129/7 males; 5 µg/ml OA: n=101/28 females and n=112/25 males; 10 µg/ml OA: n=120/5 females and n=134/7 males; 25 µg/ml OA: n= 133/14 females and n=132/5 males; Effect of OA feeding (*p=0.94)*, sex ($p=2x10^{-16}$), and OA feeding-by-sex interaction ($p=1.1x10^{-3}$); *CPH.* **e**, **f**, and **g** boxplots show quantiles with individual data points overlayed. Where relevant, statistical tests were two-sided; no multiple testing correction was applied.

a *CPH* model for lifespan of *TiGS>Octβ2R* flies

Call: coxph(formula = Surv(time = day, event = event) ~ RU486 * Sex)

	coef	Exp.coef	Pr(>|z|)
RU486 (Present)	−1.6691	0.1884	***< 2e−16
Sex (Male)	0.9775	2.6577	***3.65e−13
RU486 (Present): Sex (Male)	1.0265	2.7913	***9.84e−08

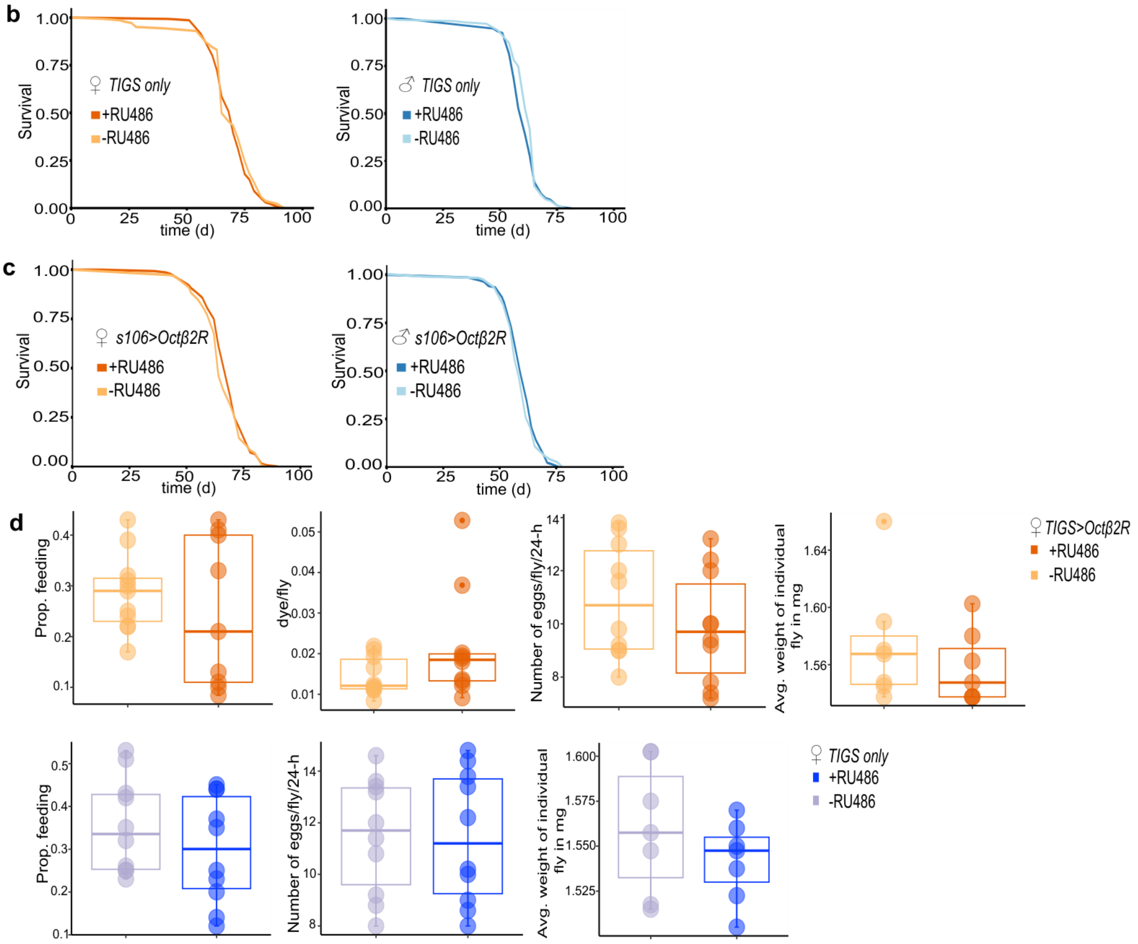

Supplementary Figure 5 *Octβ2R* - lifespan and other phenotypes.

a, *CPH* summary – for *TIGS>Octβ2R* lifespan (survival presented in **Figure 5b**). **b,** Lifespan of *TIGS*-only control flies. Induced (plus RU486): n= 139 dead/2 censored females and 130/10 males; uninduced (minus RU486): n=138/4 females and 143/4 males; effect of RU486 in females (*p=0.38*) and in males (*p= 0.17*), *log-rank test*. **c,** Lifespan of *s106>Octβ2R* flies. Induced (plus RU486): n=140/1 females and 136/3 males; uninduced (minus RU486): n=145/0 females and 145/0 males; effect of RU486 in females (*p=0.25*) and in males (*p=0.77*); *log-rank test*. **d,** Proboscis extension, food (dye) consumption assays, fecundity levels, and weight measurements of *TIGS>Octβ2R* female flies. Biologically independent samples in proboscis extension assay: n=11 (*TIGS>Octβ2R* uninduced), 9 (*TIGS>Octβ2R* induced), 10 (*TIGS only* induced and uninduced); food (dye) consumption assay: n=10; fecundity assay: n=10; weight measurements: n=7; proboscis extension assay: effect of RU486 (*p=0.44*), *GLM* with quasi-binomial distribution; food (dye) consumption assay: effect of RU486 (*p=0.15*), *t-test*; feeding proportion of *TIGS* only flies: effect of RU486 (*p=0.31*), *GLM* with quasibinomial distribution; fecundity levels of *TIGS>Octβ2R* flies: effect of RU486 (*p=0.29*), *t*-test; fecundity levels of *TIGS* only flies: effect of RU486 (*p=0.96), t*-test; weight measurements of *TIGS>Octβ2R* flies: effect of RU486 (*p=0.40*), *t-test*; weight

measurements of *TIGS* only flies: effect of RU486 (*p=0.29*), *t-test*. Boxplots show quantiles with individual data points overlayed. Where relevant, statistical tests were two-sided; no multiple testing correction was applied.

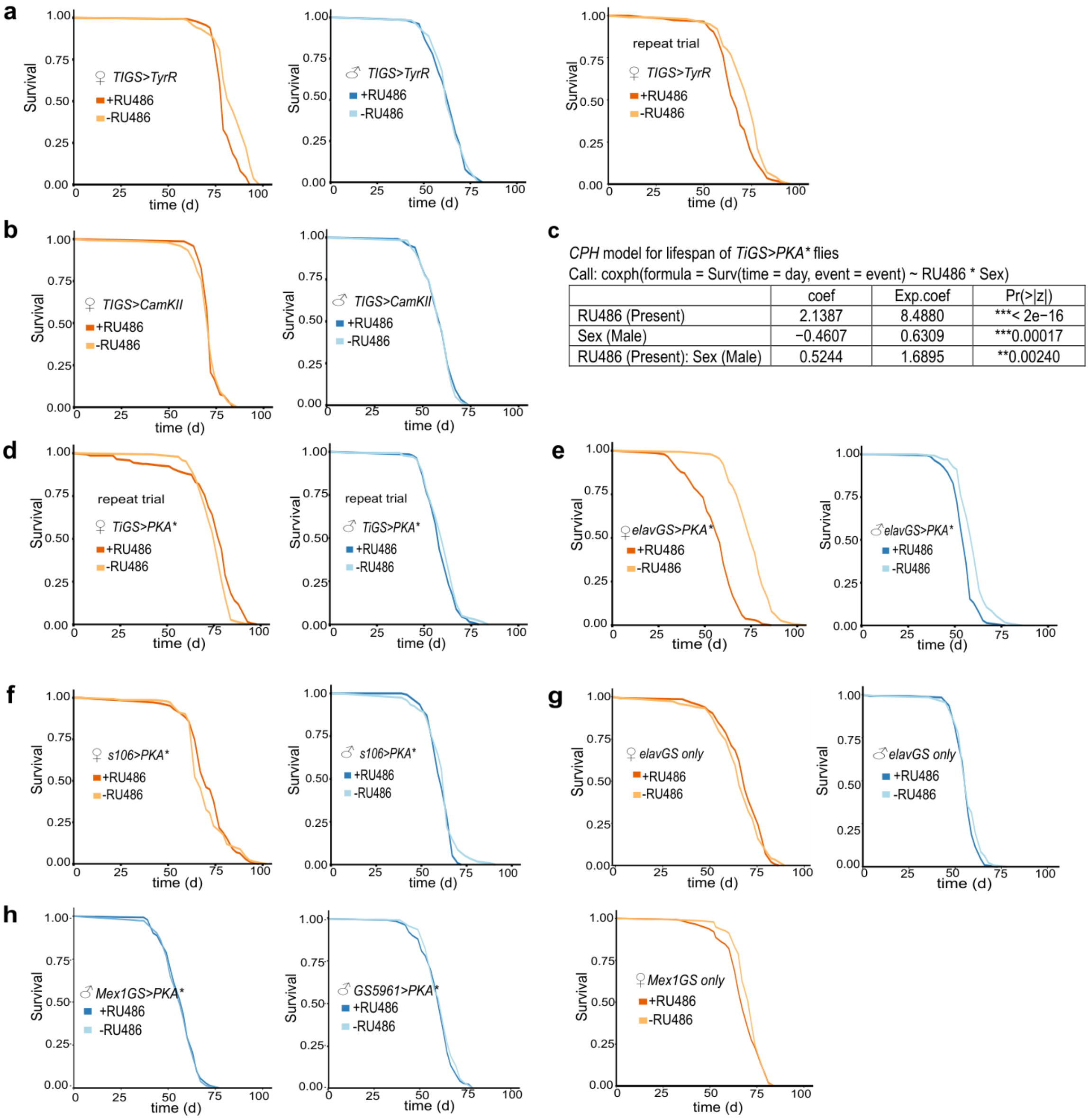

	coef	Exp.coef	Pr(>|z|)
RU486 (Present)	2.1387	8.4880	***< 2e−16
Sex (Male)	−0.4607	0.6309	***0.00017
RU486 (Present): Sex (Male)	0.5244	1.6895	**0.00240

Supplementary Figure 6 *PKA, *TyrR* and *CamKII*- lifespans.**

a, Lifespan of *TIGS>TyrR.* Induced (plus RU486): n=134/0 females and 127/0 males; uninduced (minus RU486): n=136/0 in females and 116/0 males; effect of RU486 (*p=1.7x10*$^{-8}$*),* sex (*p=2x10*$^{-16}$), and RU486-by-sex interaction (*p=2.6x10*$^{-5}$), *CPH*. Lifespan of *TIGS>TyrR* (repeat trial in female flies). Induced (plus RU486): n=112/3; uninduced (minus RU486): n=100/1; effect of RU486 (*p=1.7x10*$^{-3}$*), log-rank test*. **b,** Lifespan of *TIGS>CamKII.* Induced (plus RU486): n= 145/0 females and 118/0 males; uninduced (minus RU486): n=149/0 females and 115/0 males; effect of RU486 (*p=0.74),* sex (*p=2x10*$^{-16}$), and RU486-by-sex interaction (*p= 0.36*), *CPH*. **c,** *CPH* summary - *TIGS>PKA** flies (survival presented in **Figure 5c**). **d,** Lifespan of *TIGS>PKA** flies (repeat trial). Induced (plus RU486): n= 143/4 females and 136/3 males; uninduced (minus RU486): n=148/0 females and 143/1 males; effect of RU486 in females (*p=1.2x10*$^{-4}$) and in males (*p=0.08*); *log-rank test*. **e,** Lifespan of *elavGS>PKA** flies. Induced (plus RU486): n=142/7 females and 139/6 males;

uninduced (minus RU486): n=151/0 females and 130/20 males; effect of RU486 in females (*p*=$8.8x10^{-37}$) and in males (*p*=$6.8x10^{-9}$), *log-rank test*. **f,** Lifespan of *s106>PKA** flies. Induced (plus RU486): n= 143/4 females and 117/25 males; uninduced (minus RU486): n=141/5 females and 117/22 males; effect of RU486 in females (*p=0.23*) and in males (*p=0.11*); *log-rank test*. **g,** Lifespan of *elavGS* only flies. Induced (plus RU486): n=144/1 females and 122/7 males; uninduced (minus RU486): n=146/3 females and 130/5 males; effect of RU486 in females (*p=0.4*) and in males (*p=0.13*); *log-rank test*. **h,** Lifespan of *Mex1GS>PKA** male flies. Induced (plus RU486): n= 142/3; uninduced (minus RU486): n= 152/1; effect of RU486 (*p=0.63*), *log-rank test*. Lifespan of *GS5961>PKA** male flies. Induced (plus RU486): n= 142/1; uninduced (minus RU486): n= 142/2; effect of RU486 (*p=0.35*); *log- rank test*. Lifespan of *Mex1GS* only female flies. Induced (plus RU486): n=150/0; uninduced (minus RU486): n=137/0; effect of RU486 (*p=0.08*); *log-rank test*. Where relevant, statistical tests were two-sided; no multiple testing correction was applied.

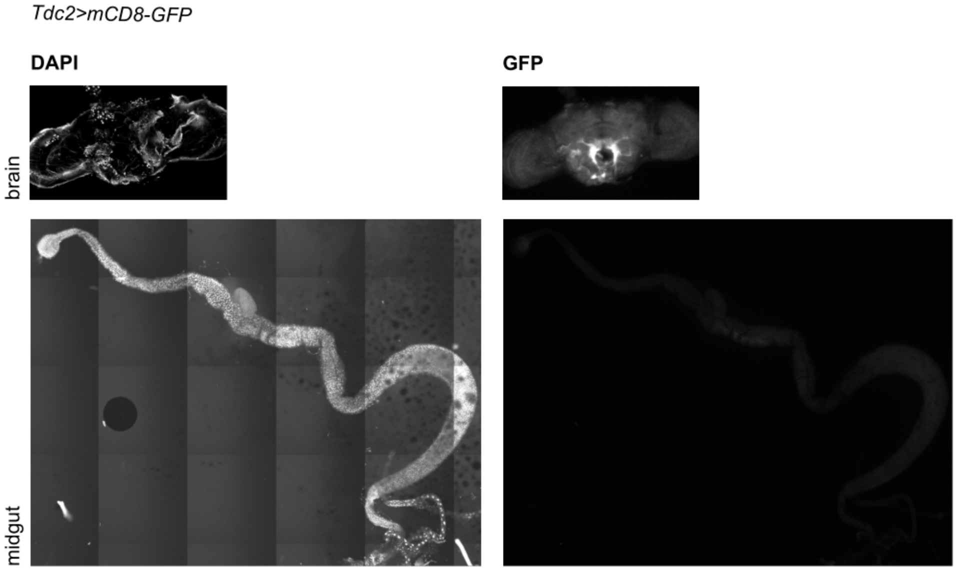

Supplementary Figure 7 Expression profile of *Tdc2*> mCD8-GFP in the fly brain and gut. The expression analysis of *Tdc2>mCD8-GFP* in fruit fly brain and gut, using confocal microscopy.

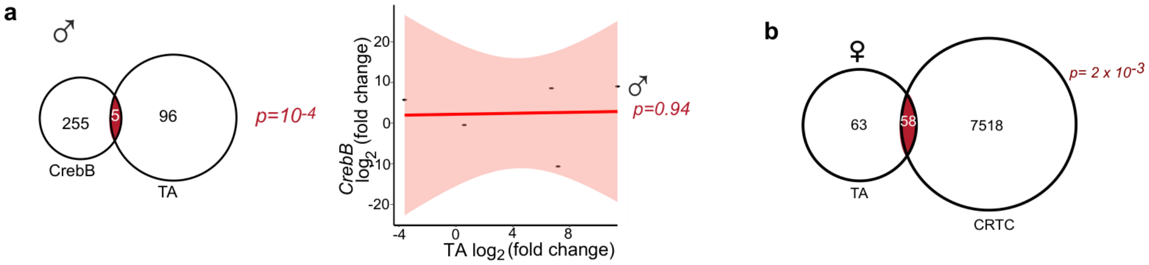

c *LM* for relative luciferase expression for TA-fed flies
Call: lm(formula = Relative_expression~ Food * Sex * Time)

Coefficients	Estimate	Std.Error	T value	Pr(>|t|)
Intercept	0.994382	0.061623	16.137	***<2e−16
Food (TA)	0.185842	0.087148	2.132	*0.0366
Sex (Male)	−0.032992	0.087148	−0.379	0.7062
Time (d15)	0.020911	0.083092	0.252	0.8021
Food (TA): Sex (Male)	0.221059	0.123246	1.794	0.0774
Food (TA): Time (d15)	−0.052816	0.117510	−0.449	0.6546
Sex (Male): Time (d15)	0.001786	0.120412	0.015	0.9882
Food (TA): Sex (Male): Time (d15)	−0.400235	0.171677	−2.331	*0.0228

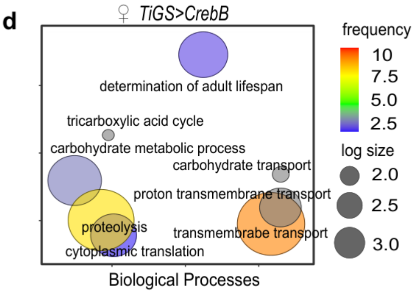

e *CPH* model for lifespan of *TiGS>CrebB*^{RNAi} flies females upon induction with RU486 andTA.
Call:coxph(formula = Surv(time = day, event = event) ~ RU486+ TA+ RU486:TA)

	coef	Exp.coef	Pr(>|z|)
RU486 (Present)	2.3099	10.0739	***< 2e−16
TA (Present)	−0.4073	0.6655	**0.00154
RU486 (Present): TA (Present)	0.5893	1.8027	**0.00115

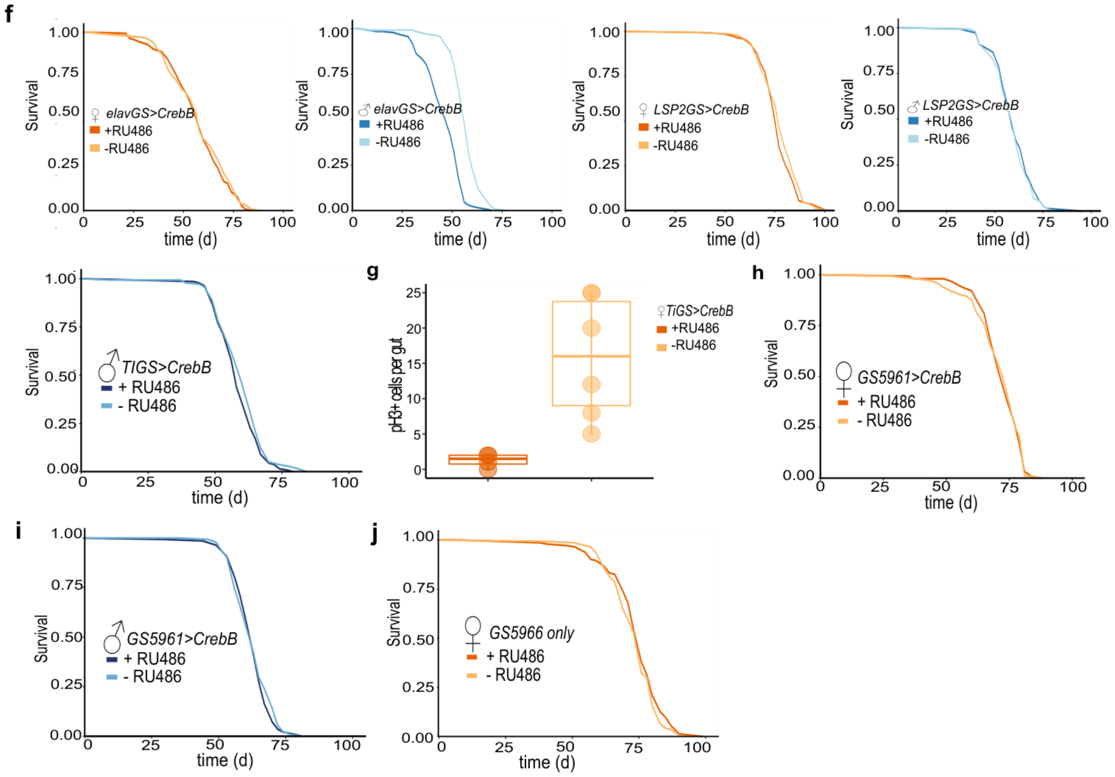

Supplementary Figure 8 *CrebB* in the gut and other tissues.
a, Overlap of transcripts differentially expressed in the gut upon *CrebB* induction (in *TIGS>CrebB*) and TA feeding in males. *P* values from a one-sided hypergeometric test. Correlation in expression upon TA feeding or *CrebB* induction for the genes within the overlap, line of best fit with 95% confidence interval (shaded). **b,** Overlap of

transcripts differentially expressed in the gut upon *CRTC* induction (*NP1>CRTC*, reanalysed from a published study) and those differentially expressed upon TA feeding in females. *P* values from a one-sided hypergeometric test. **c,** *LM* summary - relative luciferase activity upon TA feeding (data shown in **Figure 6f**). **d,** GO enrichment of biological processes for transcripts differentially regulated in guts of *TIGS>CrebB* female flies. **e,** *CPH* summary - *TIGS>CrebB*^{RNAi} flies upon induction with RU486 combined with TA feeding (survival presented in **Figure 7a**). **f**, Lifespan of *elavGS>CrebB* flies. Induced (plus RU486): n= 145 dead/1 censored females and 133/7 males; uninduced (minus RU486): n=132/9 females and 129/14 males; effect of RU486 in females (*p=0.51*) and in males ($p=2.0x10^{-37}$); *log-rank test*. Lifespan of *LSP2GS>CrebB* flies. Induced (plus RU486): n= 146/0 females and 141/2 males; uninduced (minus RU486): n=144/0 females and 144/1 males; effect of RU486 in females (*p=0.23*) and in males (*p=0.47*); *log-rank test*. Lifespan of *TIGS>CrebB* male flies. Induced (plus RU486): n= 135/2; uninduced (minus RU486): n=139/11; effect of RU486 (*p=0.12*); *log-rank test*. **g,** Number of pH3+ cells per gut of *TIGS>CrebB* 63-day old female flies. Biologically independent samples: n=5 (*TIGS>CrebB* uninduced), 4 (*TIGS>CrebB* induced); effect of RU486 (p=$9.0x10^{-3}$), *t-test*. **h,** Lifespan of *GS5961>CrebB* female flies. Induced (plus RU486): n=152/1; uninduced (minus RU486): n=161/0; effect of RU486: *p=0.71*; *log-rank test*. **i,** Lifespan of *GS5961>CrebB* male flies. Induced (plus RU486): n=140/0; uninduced (minus RU486): n=130/16; effect of RU486: *p=0.53*; *log-rank test*. **j,** Lifespan of *GS5966* only female flies. Induced (plus RU486): n=143/2; uninduced (minus RU486): n=140/0; effect of RU486 (*p=0.19*); *log-rank test*. Boxplots show quantiles with individual data points overlayed. Where relevant, statistical tests were two-sided unless otherwise noted; no multiple testing correction was applied, with the exception of transcriptomic data.

a

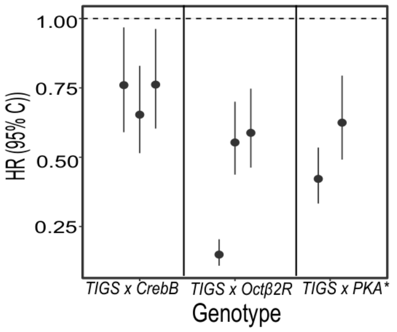

b *CPH* model for lifespan of combined induction of *CrebB* and *Octβ2R* in the gut

Call: coxph(formula = Surv(time = day, event = event) ~ RU486 * Genotype)

	coef	Exp.coef	Pr(>|z|)
RU486 (Present)	−0.40331	0.66811	***3.61e−11
Genotype (*TIGS* **vs** *TIGS>Octβ2R, TIGS>CrebB, TIGS>Octβ2R&CrebB)*	-0.01010	0.98995	0.672
Genotype (*TIGS>Octβ2R, TIGS>CrebB* **vs** *TIGS>Octβ2R & CrebB*)	0.03538	1.03601	0.298
Genotype (*TIGS>Octβ2R* **vs** *TIGS>CrebB*)	−0.04635	0.95470	0.440
RU486 (Present): Genotype (*TIGS* **vs** *TIGS>Octβ2R, TIGS>CrebB, TIGS>Octβ2R&CrebB)*	−0.13507	0.87365	***7.92e−05
RU486 (Present): Genotype (*TIGS>Octβ2R, TIGS>CrebB* **vs** *TIGS>Octβ2R & CrebB*)	−0.03780	0.96290	0.458
RU486 (Present): Genotype (*TIGS>Octβ2R* **vs** *TIGS>CrebB*)	0.04981	1.05107	0.552

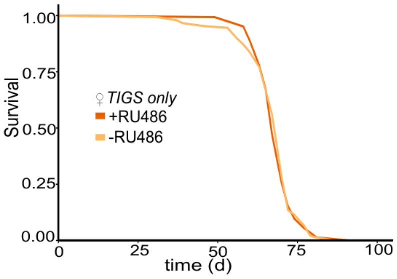

c *CPH* model for lifespan of *TiGS>Octβ2R* female flies upon induction with RU486 and TA

Call: coxph(formula = Surv(time = day, event = event) ~ RU486 + TA + RU486:TA)

	coef	Exp.coef	Pr(>|z|)
RU486 (Present)	−0.5294	0.5890	***1.28e−05
TA (Present)	−0.2744	0.7600	*0.0244
RU486 (Present): TA (Present)	0.4209	1.5233	*0.0148

d *CPH* model for lifespan of *TiGS>Octβ2R*^{RNAi} female flies upon induction with RU486 and TA.

Call: coxph(formula=Surv(time=day, event=event) ~ RU486 + TA + RU486:TA)

	coef	Ex.coef	Pr(>|z|)
RU486 (Present)	−0.1234	0.8839	0.29816
TA (Present)	−0.4012	0.6695	0.00101 **
RU486 (Present): TA (Present)	0.3687	1.4458	0.03116 *

e

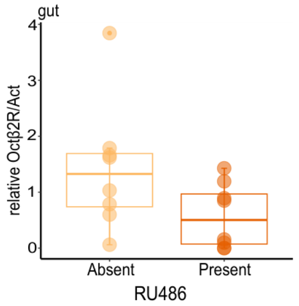

Supplementary Figure 9 *Octβ2R, PKA and *CrebB* in the gut.**

a, Hazard ratio (HR) of independent lifespan trails of *TIGS>Octβ2R*, *TIGS>PKA**, and *TIGS>CrebB* female flies, *CPH*. Mean and 95% confidence intervals are shown. **b,** *CPH* summary for the combined induction of *CrebB* and *Octβ2R* in the gut (survival

presented in **Figure 7d**). Lifespan of *TIGS* only female flies (shown below) was included in the analysis. *TIGS* only induced (plus RU486): n= 144 dead/1 censored; uninduced (minus RU486): n=148/0. To compare between the genotypes, *a priori*, orthogonal contrasts comparing the control (*TIGS only*) with three UAS-containing genotypes (*TIGS>Octβ2R*, *TIGS>CrebB* and *TIGS>Octβ2R&CrebB*); two single UAS genotypes with combined UAS genotype (*TIGS>Octβ2R* and *TIGS>CrebB* vs *TIGS>Octβ2R&CrebB*) and the two single UAS genotypes to each other (*TIGS>Octβ2R* vs *TIGS>CrebB*) were used. **c,** *CPH* summary- *TIGS>Octβ2R* flies upon induction with RU486 combined with TA feeding (survival presented in **Figure 7e**). **d,** *CPH* summary- *TiGS>Octβ2R*^{RNAi} flies upon induction with RU486 combined with TA feeding (survival presented in **Figure 7f**). **e,** qPCR quantification of *Octβ2R* mRNA levels in 7-day old *TiGS>Octβ2R*^{RNAi} female guts at the indicated time. n=8 biologically independent samples; significant effect of RU486 (p=*0.045*), *one-tailed t-test*. Boxplots show quantiles with individual data points overlayed. Where relevant, statistical tests were two-sided unless otherwise noted; no multiple testing correction was applied.

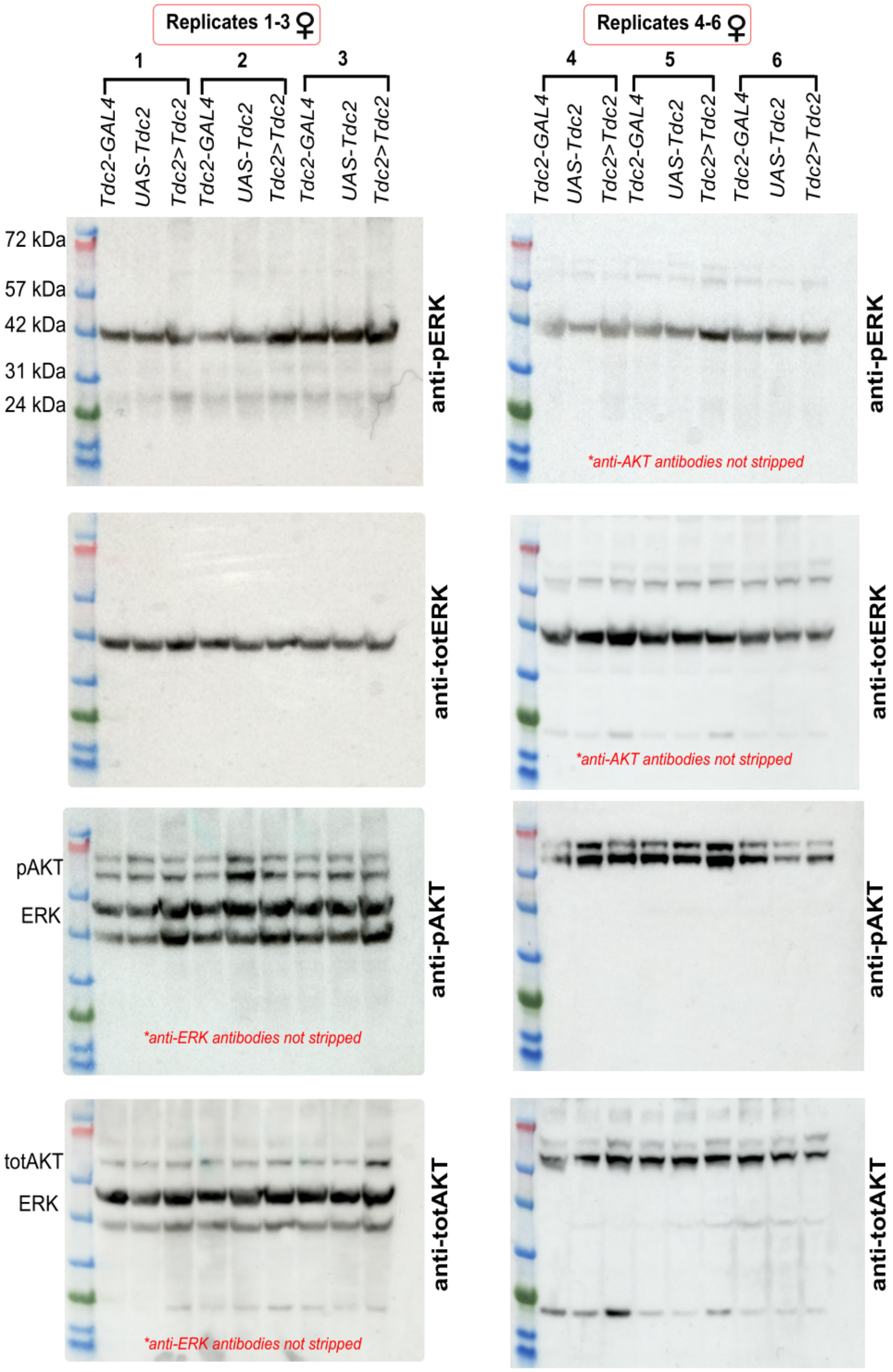

Supplementary Figure 10 Western blots in *Tdc2>Tdc2* female flies. The images show full blots (uncropped) and are unprocessed 8 bit JPEG images provided by the imaging software. Note that 16 bit TIFF images obtained at the same time were used for quantification. Uncropped western blot images for the quantification of pAKT, AKT, pERK and ERK in *Tdc2>Tdc2* female flies. n=6 independent biological samples per genotype, analysed in two independent blots.
