# Supplementary material for: ß-adrenergic-like signalling engages CrebB in Drosophila gut to promote female longevity
Source: Nat Commun. 2026 Apr 2;17:4844. doi: 10.1038/s41467-026-71341-y (PMC13222880; doi:10.1038/s41467-026-71341-y)
Supplement: Supplementary file 2 — Reporting Summary [file 41467_2026_71341_MOESM2_ESM.pdf]

Corresponding author(s): Nazif Alic

Last updated by author(s): 2026/02/05

Reporting Summary

Nature Portfolio wishes to improve the reproducibility of the work that we publish. This form provides structure for consistency and transparency in reporting. For further information on Nature Portfolio policies, see our Editorial Policies and the Editorial Policy Checklist.

Statistics

For all statistical analyses, confirm that the following items are present in the figure legend, table legend, main text, or Methods section.

n/a	Confirmed	
☐	☒	The exact sample size (*n*) for each experimental group/condition, given as a discrete number and unit of measurement
☐	☒	A statement on whether measurements were taken from distinct samples or whether the same sample was measured repeatedly
☐	☒	The statistical test(s) used AND whether they are one- or two-sided<br>*Only common tests should be described solely by name; describe more complex techniques in the Methods section.*
☐	☒	A description of all covariates tested
☐	☒	A description of any assumptions or corrections, such as tests of normality and adjustment for multiple comparisons
☐	☒	A full description of the statistical parameters including central tendency (e.g. means) or other basic estimates (e.g. regression coefficient) AND variation (e.g. standard deviation) or associated estimates of uncertainty (e.g. confidence intervals)
☐	☒	For null hypothesis testing, the test statistic (e.g. *F*, *t*, *r*) with confidence intervals, effect sizes, degrees of freedom and *P* value noted<br>*Give P values as exact values whenever suitable.*
☒	☐	For Bayesian analysis, information on the choice of priors and Markov chain Monte Carlo settings
☒	☐	For hierarchical and complex designs, identification of the appropriate level for tests and full reporting of outcomes
☒	☐	Estimates of effect sizes (e.g. Cohen's *d*, Pearson's *r*), indicating how they were calculated

Our web collection on statistics for biologists contains articles on many of the points above.

Software and code

Policy information about availability of computer code

Data collection	N/A
Data analysis	R (v4.4.1) and libraries: "ordinal": ‘2023.12.4.1’; "DeSeq2": ‘1.44.0’; "dplyr": ‘1.1.4’, "ggplot2": ‘3.5.1’; "tidyverse": ‘2.0.0’; "survminer": ‘0.5.0’; "survival": ‘3.6.4’; "tximportData": ‘1.32.0’; "tximport": ‘1.32.0’; "readr": ‘2.1.5’; "IHW": ‘1.32.0’; "VennDiagram": ‘1.7.3’; "pheatmap": ‘1.0.12’. ImageJ (v1.54d).

For manuscripts utilizing custom algorithms or software that are central to the research but not yet described in published literature, software must be made available to editors and reviewers. We strongly encourage code deposition in a community repository (e.g. GitHub). See the Nature Portfolio guidelines for submitting code & software for further information.

Data

Policy information about availability of data

All manuscripts must include a data availability statement. This statement should provide the following information, where applicable:

- Accession codes, unique identifiers, or web links for publicly available datasets
- A description of any restrictions on data availability
- For clinical datasets or third party data, please ensure that the statement adheres to our policy

Raw RNA-Seq data have been submitted to GEO, accession numbers: GSE276897 [https://www.ncbi.nlm.nih.gov/geo/query/acc.cgi?acc=GSE276897] and GSE276880 [https://www.ncbi.nlm.nih.gov/geo/query/acc.cgi?acc=GSE276880]. Data previously deposited in GEO was also used, accession number: GSE185159 [https://www.ncbi.nlm.nih.gov/geo/query/acc.cgi?acc=GSE185159]. The analysis results are given in Supplementary Data. Uncropped images of the western blots

are available in Supplementary Figure 10. All data supporting the conclusion of the paper are available within the Supplementary Data. Source data are provided with this study.

Research involving human participants, their data, or biological material

Policy information about studies with human participants or human data. See also policy information about sex, gender (identity/presentation), and sexual orientation and race, ethnicity and racism.

Reporting on sex and gender	N/A
Reporting on race, ethnicity, or other socially relevant groupings	N/A
Population characteristics	N/A
Recruitment	N/A
Ethics oversight	N/A

Note that full information on the approval of the study protocol must also be provided in the manuscript.

Field-specific reporting

Please select the one below that is the best fit for your research. If you are not sure, read the appropriate sections before making your selection.

[x] Life sciences [ ] Behavioural & social sciences [ ] Ecological, evolutionary & environmental sciences

For a reference copy of the document with all sections, see nature.com/documents/nr-reporting-summary-flat.pdf

Life sciences study design

All studies must disclose on these points even when the disclosure is negative.

Sample size	Sample size was determined based on previous, similar experiments.
Data exclusions	Flies accidentally lost or killed during lifespan assays were censored. In luciferase assays, to protect against outliers, two highest and two lowest measurements from each condition/time point were excluded from the analysis of whole fly data. In climbing assays, flies whose height could not be determined from the still image were not included in the analysis. Details are provided in the manuscript.
Replication	All attempts at replication are included in the manuscript. All replication attempts were successful.
Randomization	Flies were randomly allocated to treatment groups in a way that avoids batching artefacts.
Blinding	Researchers were blinded to the experimental conditions in the proboscis extension assays. They were not blinded in any other assay.

Reporting for specific materials, systems and methods

We require information from authors about some types of materials, experimental systems and methods used in many studies. Here, indicate whether each material, system or method listed is relevant to your study. If you are not sure if a list item applies to your research, read the appropriate section before selecting a response.

Materials & experimental systems

n/a	Involved in the study	
[ ]	[x]	Antibodies
[x]	[ ]	Eukaryotic cell lines
[x]	[ ]	Palaeontology and archaeology
[ ]	[x]	Animals and other organisms
[x]	[ ]	Clinical data
[x]	[ ]	Dual use research of concern
[x]	[ ]	Plants

Methods

n/a	Involved in the study	
[x]	[ ]	ChIP-seq
[x]	[ ]	Flow cytometry
[x]	[ ]	MRI-based neuroimaging

Antibodies

Antibodies used	Cell Signaling: anti-phospho-H3 (#9701), anti-total-ERK (#4695), anti-phospho-ERK (#4370), anti-total-AKT (#9272), or anti-phospho-AKT (#9271) . Antibodies were used as per manufacturer's instructions (1:1000 dilution). 2ry antibodies were HRP-conjugates from Abcam (1:10000,ab6721) .

Validation	The antibodies were validated by the manufacturer. All have been widely used e.g. references Slack at al 2015, Filer et al. 2017, Martinez-Corrales et al 2020 in the manuscript.

Animals and other research organisms

Policy information about studies involving animals; ARRIVE guidelines recommended for reporting animal research, and Sex and Gender in Research

Laboratory animals	The outbred wild-type stock was obtained in present-day Benin in 1970s and has been maintained in population cages to preserve fecundity and lifespan at levels of freshly caught flies. The w1118 or v1 mutation was incorporated into this background by backcrossing to allow tracking of genetic constructs. Outbred w1118 population was cleared of Wolbachia by tetracycline treatment several years ago; all experimental flies were negative for Wolbachia. The population cages were obtained from L. Partridge.<br>Transgenic lines were obtained from Bloomington Drosophila Stock Centre or Vienna Drosophila Resource Center except:UAS-Tbh (M. Monastirioti), TIGS (S. Pletcher), S1106 and elavGS (L. Partridge), Mex1GS and Lsp2GS (J.-R. Martin), GS5961/66 (B. Ohlstein)<br>Transgenes: UAS-Tdc2 (P{UAS-Tdc2.C}, ref.19, RRID:BDSC_9316), UAS-Tbh (ref.35), UAS-Octβ2R (P{UAS-Octβ2R.S}, deposited in Bloomington Drosophila Stock Centre (BDSC) by Schwarzel, M., RRID:BDSC_78806), UAS-PKA* (UAS-mc*, ref.50), UAS-TyrR (P{UAS-TyrR.H}, ref.86, RRID:BDSC_67128), UAS-CaMKII (P{UAS-CaMKII.R3}, ref.87, RRID:BDSC_29662), UAS-CrebB (P{UAS-CrebB-17A-a.cor}, ref.88, RRID:BDSC_9233), 5xCRE-Luc (P{5xCRE-LUC}, deposited in BDSC by Fayyazuddin, A., used in ref.62, RRID:BDSC_79016), UAS-CrebBRNAi (P{TRiP.HMJ30249}, ref.89, RRID:BDSC_63681), UAS-Octβ2RRNAi (P{GD2954} from Vienna Drosophila Resource Center (VDRC), used in ref.69), UAS-mCD8-GFP and drivers: Tdc2-GAL4 (P{Tdc2-GAL4.C}, ref.19, RRID:BDSC_9313), Tbh-GAL4 (P{GMR76H05-GAL4}, ref.36, RRID:BDSC_45904), TIGS and S1106 (ref.45), GS5961 and GS5966 (ref.53), Mex1GS (ref.52) LSP2GS (ref.65) and elavGS (ref.51) were backcrossed at least six times into the outbred background. The transgenes were kept in large populations and frequently outcrossed back into the outbred population to avoid bottlenecks and genetic drift. References given in the manuscript.
Wild animals	N/A
Reporting on sex	Sex of the animals is reported in the manuscript. Most often, both sexes were used, separately. The combinations of sex, genotype and age in the experiments are too complex and varied to report here - please see manuscript for details.
Field-collected samples	N/A
Ethics oversight	Ethical approval was not required.

Note that full information on the approval of the study protocol must also be provided in the manuscript.

Plants

Seed stocks	N/A
Novel plant genotypes	N/A
Authentication	N/A
